# Supplementary material for: SCODE: an efficient regulatory network inference algorithm from single-cell RNA-Seq during differentiation
Source: Bioinformatics. 2017 Apr 4;33(15):2314–21. doi: 10.1093/bioinformatics/btx194 (PMC5860123; doi:10.1093/bioinformatics/btx194)
Supplement: Supplementary Data [file btx194_supplementary_text.pdf]

Supplementary Text for SCODE: An efficient regulatory network inference algorithm from single-cell RNA-Seq during differentiation

Hirotaka Matsumoto, Hisanori Kiryu, Chikara Furusawa,
Minoru S.H. Ko, Shigeru B.H. Ko, Norio Gouda,
Tetsutaro Hayashi, and Itoshi Nikaido

January 26, 2017

1 Optimization algorithm of SCODE

The detailed procedure of parameter optimization in SCODE is as follows.

1. Initialize a diagonal matrix $\mathbf{B}$ randomly, and set $\hat{\mathbf{B}}$ to $\mathbf{B}$.

2. Generate $\mathbf{Z}^{(e)}$ from the ODE of $\mathbf{z}$ determined from $\mathbf{B}$. (The initial value of $\mathbf{z}_i$ ($i \in [1, D]$) is set to 1. We add uniform random values $\epsilon \in [-0.001, 0.001]$ to each element of $\mathbf{Z}^{(e)}$ to avoid overfitting.)

3. Optimize $\mathbf{W}$ based on linear regression, and calculate $\mathrm{RSS}(\mathbf{B}, \mathbf{W})$.

4. If $\mathrm{RSS}(\mathbf{B}, \mathbf{W}) < \mathrm{RSS}(\hat{\mathbf{B}}, \hat{\mathbf{W}})$, we update $\hat{\mathbf{B}}$ with $\mathbf{B}$ and $\hat{\mathbf{W}}$ with $\mathbf{W}$.

5. Set $\mathbf{B}$ to $\hat{\mathbf{B}}$.

6. Sample $i \in [1, D]$ uniformly, and sample $\mathbf{B}_{ii} \in [b_{\min}, b_{\max}]$ uniformly.

7. Return to step 2 unless the number of iterations reaches the limit. (In this study, we used 100 as the maximum number of iterations.)

8. After iterative optimization, $\mathbf{A}$ is inferred from $\mathbf{A} = \hat{\mathbf{W}}\hat{\mathbf{B}}\hat{\mathbf{W}}^{+}$.

2 Reconstruction of expression dynamics

In the main text, we validated that SCODE can successfully reconstruct observed expression dynamics for some TFs in Data1. In this section, we present our investigation of the global differences between reconstructed dynamics and observed data for all TFs and datasets. To evaluate the differences between

reconstructed dynamics and observed data, we defined the *absolute residual* as the absolute value of the difference between the observed expression and the value of the reconstructed dynamics at the corresponding time point.

Fig 1(a) shows the histogram of expression data ($\mathbf{X}^{(e)}$) for each dataset. The scales of expression values differ among datasets owing to differences such as standardization methods. Fig 1(b) is the histogram of mean absolute residuals of 100 TFs. For every dataset, the histograms of mean absolute residuals exhibit unimodal distributions, and there are no significant outlier TFs. The means of expression are about 0.49, 2.9, and 2.7 for Data1, Data2, and Data3, respectively, and the means of mean absolute residuals are about 0.16, 1.7, and 1.2, respectively. The ratios of the mean of mean absolute residuals to the that of expression values are about 0.32, 0.59, and 0.44, and therefore we concluded that the residuals are small compared with the scale of the expression values and the dynamics of all TFs can be reconstructed accurately.

This ratio is largest for Data2. The histogram of expression in Data2 shows that there are many zero expression data points, and some of them must be due to dropout events, which results in zero-inflated data. Therefore, the ratio for Data2 is largest because the zero-inflated data produced a large absolute residual. Although such zero-inflated expression data have a negative influence on parameter estimation, our algorithm was still able to estimate the appropriate dynamics. This is because $\mathbf{W}$ can estimated appropriately based on other observed data in linear regression. Thus, our algorithm might be robust to such zero-inflated data that often occur in single-cell sequencing data.

Next, we investigated the influence of variance in expression on the absolute residuals. We compared the variance with the mean absolute residuals (see Fig 2). In general, the mean absolute residuals become large in accordance with the variance, and there are no TFs for which the mean absolute residual is large compared with the variance. Therefore, the variance in expression is the main cause of the large absolute residuals, and there are no TFs whose reconstructed dynamics significantly stray from the observed data.

On the other hand, there are some TFs whose mean absolute residuals are small in spite of large variance. In these cases, the large variance is caused by significant expression changes throughout differentiation, and the reconstructed dynamics fit such significant expression changes. Interestingly, *Gata4* and *Gata6*, which significantly influence differentiation [1], and *Sox2* and *Nanog*, which are pluripotency TFs, are among such TFs in Data1 and Data3. In addition, *Ascl1*, which is overexpressed to transform MEFs to myocytes, is also among such TFs in Data2 [2]. These results suggest that such TFs might be strongly related to the differentiation process, and we might be able to infer the drivers of differentiation through this analysis.

3 Validation with small-scale experimental studies

We compared the estimated network of SCODE of Data3 with TRRUST, which is a human transcriptional regulatory network database based on small-scale experimental studies [3]. Firstly, we extracted the regulatory relationships from TRRUST so that both of regulator and target are contained in 100 TFs. We removed relationships whose annotation of regulatory type contain unknown or both of activation and repression. In consequence, 13 relationships among 16 TFs were obtained (Fig 3(a)). Next, we extracted the network of the 16 TFs from estimated network of SCODE (optimized $\mathbf{A}$) (Fig 3(b)). To obtain reliable network, we removed the edges which satisfy $-0.5 < \mathbf{A}_{ij} < 0.5$. As a result, the experimentally validated positive regulation from *GATA3* to *ZEB1* [4] was detected by SCODE. Because *GATA3* is a known marker for the differentiation of Data3, this regulatory relationship will be effectual in the differentiation. On the other hand, the network of SCODE did not contain any regulation from *POU5F1* regardless of many regulation in TRRUST. This is because the regulation from *POU5F1* are mainly investigated by the study of pluripotency and self-renewal of ES cells [5], and such regulation will not work in differentiating cells.

In summary, SCODE succeeded to detect one validated and convincing regulatory relationship. The regulatory relationships which are validated with small-scale experimental study are limited, and bioinformatics approaches including our method are necessary to reveal comprehensive transcriptional regulatory network.

4 Network analysis

As in the main text, we defined the threshold as the value of the 1000-th largest absolute $\mathbf{A}$ value and counted the number of positive and negative edges of each TF for each dataset. Fig 4 shows the number of edges for each TF in decreasing order. About 39, 45, and 51% of edges are included in the top 10 TFs for each dataset, and this result suggests that a small number of factors mainly regulate differentiation.

Interestingly, TFs tend to have either positive or negative edges in Data1 and Data3. In contrast, the ratio of the number of positive edges to that of negative edges is balanced in Data2. Data1 and Data3 represent differentiation from ES cells, and Data2 is scRNA-Seq data representing direct reprogramming from MEFs; this difference might reflect the dissimilarity between the cellular state transition systems.

References

[1] D. Shimosato, M. Shiki, and H. Niwa. Extra-embryonic endoderm cells derived from ES cells induced by GATA factors acquire the character of XEN cells. *BMC Dev. Biol.*, 7:80, 2007.

[2] B. Treutlein et al. Dissecting direct reprogramming from fibroblast to neuron using single-cell RNA-seq. *Nature*, 534(7607):391–395, Jun 2016.

[3] H. Han et al. TRRUST: a reference database of human transcriptional regulatory interactions. *Sci Rep*, 5:11432, Jun 2015.

[4] W. Sun, S. Yang, W. Shen, H. Li, Y. Gao, and T. H. Zhu. Identification of DeltaEF1 as a novel target that is negatively regulated by LMO2 in T-cell leukemia. *Eur. J. Haematol.*, 85(6):508–519, Dec 2010.

[5] Y. Babaie, R. Herwig, B. Greber, T. C. Brink, W. Wruck, D. Groth, H. Lehrach, T. Burdon, and J. Adjaye. Analysis of Oct4-dependent transcriptional networks regulating self-renewal and pluripotency in human embryonic stem cells. *Stem Cells*, 25(2):500–510, Feb 2007.

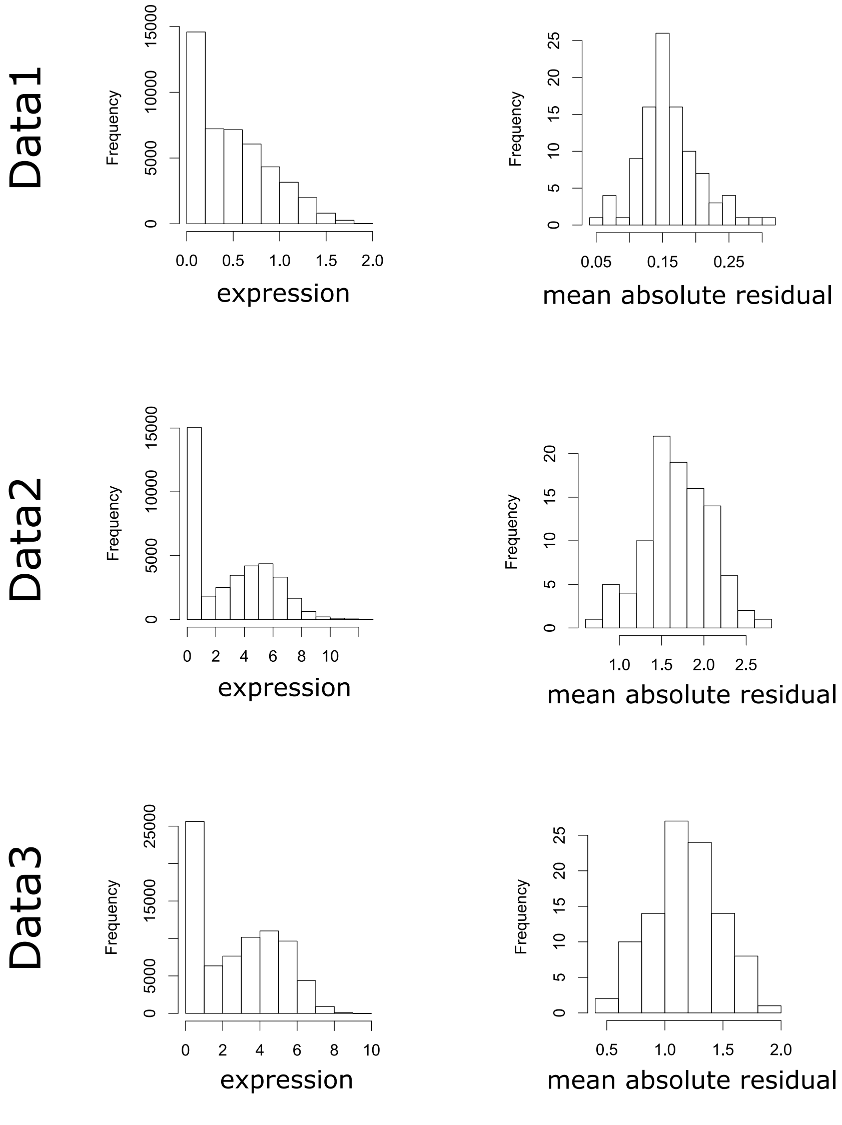

Figure 1: (a) Histogram of expression data for each dataset. (b) Histogram of the mean absolute residual of 100 TFs.

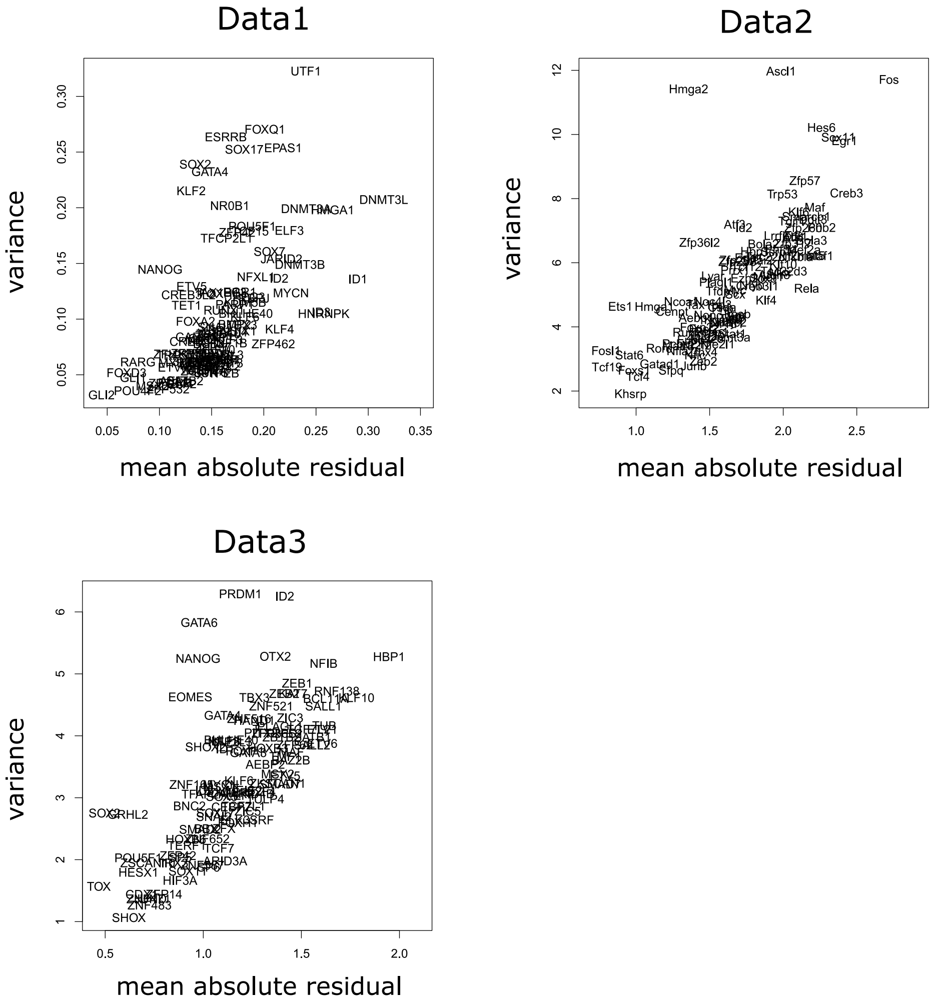

Figure 2: Comparison of variance and mean absolute residual for each TF.

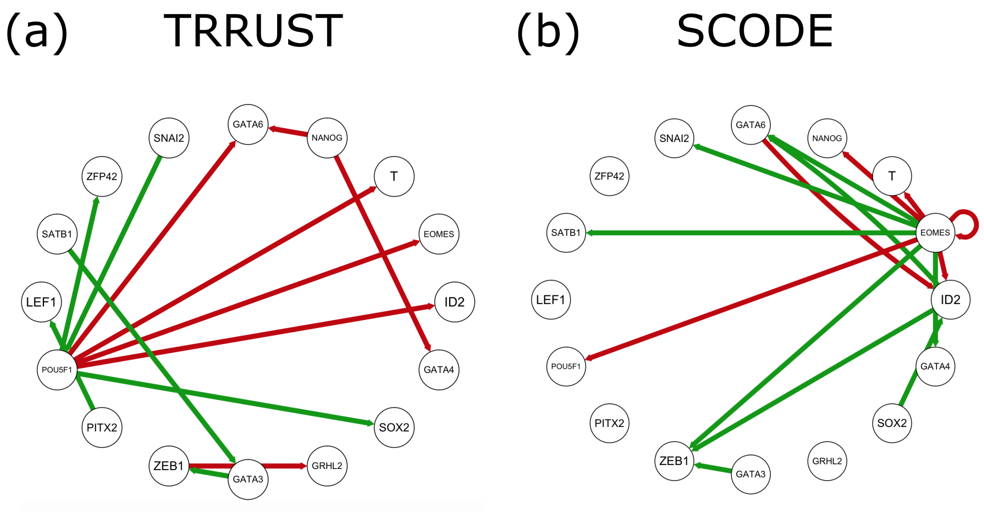

Figure 3: The regulatory network of TRRUST (a) and SCODE (b). The green and red arrows represent activation and repression, respectively.

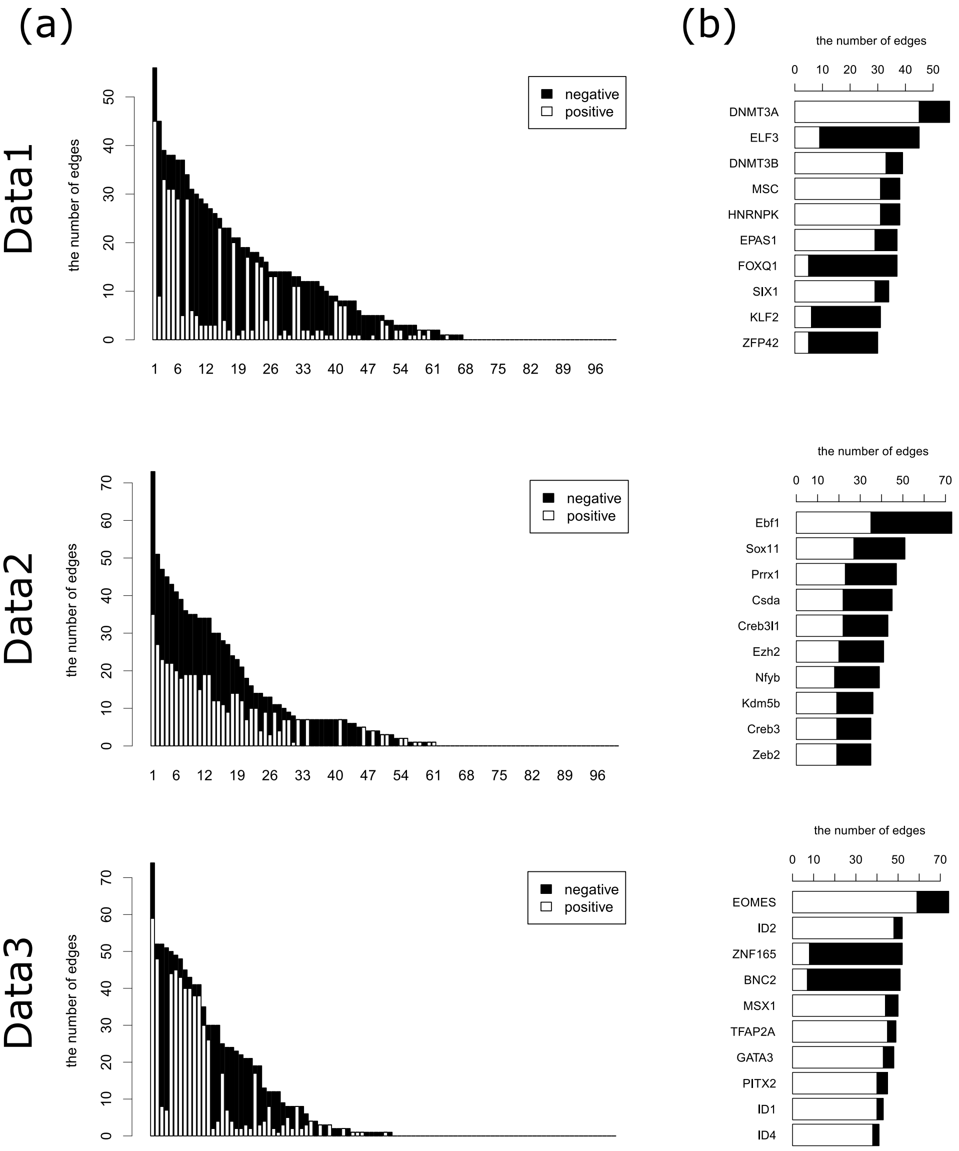

Figure 4: (a) Bar graph of the positive and negative edges of each TF in decreasing order. (b) Bar graph only for the top 10 TFs.
